# Large Aberration Correction by Magnetic Fluid Deformable Mirror with Model-Based Wavefront Sensorless Control Algorithm

**DOI:** 10.3390/ijms20153697

**Published:** 2019-07-28

**Authors:** Xiang Wei, Yuanyuan Wang, Zhan Cao, Dziki Mbemba, Azhar Iqbal, Zhizheng Wu

**Affiliations:** 1 Department of Precision Mechanical Engineering, Shanghai University, Shanghai 200444, China; 2 Dunlap Institute for Astronomy and Astrophysics, University of Toronto, Toronto, ON M5S 3H4, Canada

**Keywords:** magnetic fluid, deformable mirror, wavefront sensorless, adaptive optics

## Abstract

Magnetic fluid is a stable colloidal suspension of nano-sized, single-domain ferri/ferromagnetic particles dispersed in a liquid carrier. The liquid can be magnetized by the ferromagnetic particles aligned with the external magnetic field, which can be used as a wavefront corrector to correct the large aberrations up to more than 100 µm in adaptive optics (AO) systems. Since the measuring range of the wavefront sensor is normally small, the application of the magnetic fluid deformable mirror (MFDM) is limited with the WFS based AO system. In this paper, based on the MFDM model and the relationship between the second moment (SM) of the aberration gradients and the far-field intensity distribution, a model-based wavefront sensorless (WFSless) control algorithm is proposed for the MFDM. The correction performance of MFDM using the model-based control algorithm is evaluated in a WFSless AO system setup with a prototype MFDM, where a laser beam with unknown aberrations is supposed to produce a focused spot on the CCD. Experimental results show that the MFDM can be used to effectively compensate for unknown aberrations in the imaging system with the proposed model-based control algorithm.

## 1. Introduction

Magnetic fluid is a stable colloidal suspension of nano-sized (about 10 nm in diameter), single-domain ferri/ferromagnetic particles dispersed in a liquid carrier. In the presence of an external magnetic field, the ferromagnetic particles align with the field, and the liquid becomes magnetized. The reflective surface can be deformed with the magnetic fluid using a locally applied magnetic field and thus serves as a deformable mirror in adaptive optics (AO) systems [1,2,3]. Conventional AO systems utilize spatial light modulators (SLMs) or solid deformable mirrors (DM) as a wavefront corrector (WFC). However, the SLMs are limited by the relatively small magnitude of correction that they can provide, usually in the range of a few micrometers [4,5] and they cannot transmit the beams with wavelengths longer than 1.6 µm [6]. Solid deformable mirrors offer small inter-actuator strokes and the maximum deflection magnitudes are normally limited to tens of micrometers [7,8], which cannot be used to correct the large aberrations. For example, the defective aberrations of the rotating liquid telescope could reach a peak-to-valley amplitude of more than 180 µm [9,10]. The proposed magnetic fluid deformable mirror (MFDM) in this paper consists of the magnetic fluid, miniature electromagnetic coils, Maxwell coil, and a thin film of a reflective material. The magnetic fluid can be deformed by the perturbed magnetic field generated by the miniature electromagnetic coils placed underneath the fluid layer. In order to linearize the response of the mirror surface, an external strong and uniform magnetic field produced by the Maxwell coil is superposed to the magnetic field of the actuators. In addition, magnetic fluids can be coated with silver liquid-like thin films to improve the reflectance [11,12]. Due to the property of free surface movement, the MFDM can easily produce strokes of more than 100 µm both for the single actuator or inter-actuators. Furthermore, it also has other advantages such as the smooth continuous mirror surface, low manufacturing cost and easy scalability [2,3]. In papers [13,14], the traditional multi-input multi-output (MIMO) PID controller and the mixed sensitivity $H_{\infty }$ controller have been used to control the mirror surface of MFDM, where the performance of the designed control loops are based on the accurate real-time measurement of the distorted wavefront using a Shack-Hartmann wavefront sensor (SHWS). However, the measuring range of the most widely used wavefront sensor in AO systems is normally smaller than 60 µm [15], which could limit the use of the MFDM in those applications with large aberrations. In order to extend the applications of MFDM for the large aberration correction, in this paper, a model-based wavefront sensorless (WFSless) control method for MFDM has been developed. WFSless AO systems operate by sequentially modulating the WFC and maximizing a feedback signal according to particular optimization algorithms. The typical WFSless model-free control methods need many intensity measurements or evaluations of the metric function, which limit the convergence rate of AO systems and may drop into local optima [16,17,18]. The model-based control methods often use a certain mapping relationship between the performance index and the control input to design the control algorithms, such as the modal approach [19], the nonlinear model identification approach [20] and geometric optical principles [21,22]. In this paper, based on the established MFDM model, a model-based WFSless AO control algorithm is proposed. Since the control approach only uses Z+1 photodetector measurement for the Z aberration modes as the predetermined bias functions, the correction capability and the convergence speed of the AO system are improved. 

In the following, the surface dynamic model of the magnetic fluid deformable mirror is first established, then based on the mapping relationship between the second moment (SM) of the aberration gradients and the far-field intensity distribution, the iterative control algorithm is presented. Finally, the correction performance of the MFDM to the unknown aberrations is evaluated in a WFSless AO setup system with the proposed WFSless control algorithm.

## 2. Modeling of Magnetic Fluid Deformable Mirror

**Figure 1:**
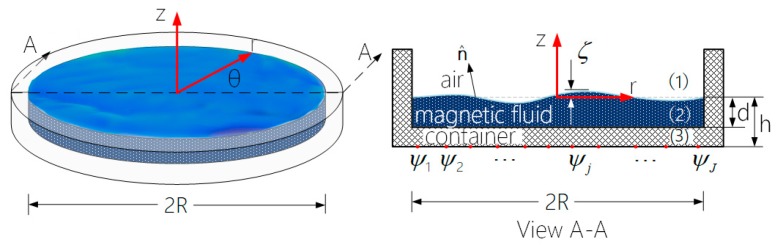
Geometric representation of a circular magnetic fluid deformable mirror.

As shown in Figure 1, in a circular coordinate system, the shape of the mirror is described by the deflection ζ(r,θ,t) of the deformed surface as measured with respect to a point (r,θ) in the horizontal plane. The deflection is produced by the cumulative magnetic field generated by the array of miniature electromagnetic coils located underneath the magnetic fluid layer. The magnetic field generated by any given coil j, j=1,2,…,J centered at the horizontal location $\left(r_{j},\theta_{j}\right)$, is idealized as that of a point source of magnetic potential $\psi_{j}\left(t\right)$.

The deflection of the free surface of a magnetic fluid results from the fluid flow induced by the applied magnetic field. The fluid flow is governed by the fundamental principles of fluid dynamics appropriately modified to account for the effects of the magnetic field. The equations governing the fluid field, derived from the principles of conservation of mass and momentum, respectively, are as follows [23]:

(1) $$\nabla \cdot \tilde{V}=0$$

(2) $$\rho \left(\frac{\partial \tilde{V}}{\partial t}+\tilde{V}\cdot \nabla \tilde{V}\right)=-\nabla \left(p+p_{s}+p_{m}\right)+\eta \nabla^{2}\tilde{V}+\rho g+\mu_{0}M\nabla H$$

where $\tilde{V}$ is the velocity of the fluid; p, $p_{s}$ and $p_{m}$ are, respectively, the thermodynamic, magnetostrictive, and fluid-magnetic pressures; ρ and η are the density and viscosity of the fluid; g is the gravitational acceleration; $\mu_{0}$ is the magnetic permeability of free space; and M and H are the magnitudes of the magnetization vector M and the magnetic field vector H, respectively.

The magnetic field itself is governed by Maxwell’s equations. Since the magnetic field of the micro coils is idealized as that of point sources of magnetic potential located at the fluid domain boundary, a current-free electromagnetic field can be assumed. Using this assumption and further assuming that the displacement currents in the fluid are negligible, Maxwell’s equations can be written as follows:

(3) ∇×H=0, ∇⋅B=0

where B is the magnetic flux density, which is related to the magnetic field H and the magnetization M by the following constitutive relationship:

(4) $$B=\mu H=\mu_{0}\left(H+M\right)$$

where μ is the magnetic permeability of the magnetic fluid. Assuming the magnetic fluid is linearly magnetized by the applied field, the magnetization vector M can be written as

(5) M=χH

where $\chi =\left(\left(\mu /\mu_{0}\right)-1\right)$ is the susceptibility of the fluid, which is considered to be constant. 

The velocity field $\tilde{V}$ can be written in terms of a scalar potential Φ(x,y,z,t) as

(6) $$\tilde{V}=-\nabla \Phi$$

such that Φ obeys the Laplace equation

(7) $$\nabla^{2}\Phi =0\;\mathrm{for}\;-h<z<\zeta$$

The magnetic field H can be written in terms of a scalar potential Ψ(x,y,z,t) as

(8) H=−∇Ψ

such that Ψ obeys the Laplace equation

(9) $$\nabla^{2}\Psi =0\;\mathrm{for}\;-h<z<\zeta$$

Since the applied magnetic field is not expected to induce any volume change in the fluid, the magnetostrictive pressure $p_{s}$ can be ignored. Moreover, the assumption that the magnetization of the fluid depends on the magnetic field only allows the magnetic pressure term $-\nabla p_{m}$ and the magnetic body force $\mu_{0}M\nabla H$ in Equation (2) to cancel each other. Using these simplifications, the surface dynamic can be written as

(10) $$-\rho \frac{\partial \Phi }{\partial t}+\rho g\zeta -\frac{\mu_{0}\chi }{2}\left(\nabla \Psi \cdot \nabla \Psi +\chi {\left(\nabla \Psi \cdot \hat{n}\right)}^{2}\right)+p^{a}+2\sigma \kappa =0\;\mathrm{at}\;z=\zeta$$

where p is the fluid pressure immediately below the interface, $p^{a}$ is the air pressure immediately above the interface, 2σκ is the capillary pressure expressed as a function of the coefficient of surface tension σ and the surface curvature κ, and $\hat{n}$ is a unit vector directed normal to the surface.

The set of Equation (7), (9), (10) can be solved to obtain the three unknowns ζ, Φ, Ψ. However, Equation (10) above is nonlinear in Ψ, therefore, linear solution methods cannot be applied to it. This complication can be circumvented by introducing a large uniform magnetic field with a constant flux density $B_{0}$ superimposed on the input field generated by the array of microcoils. 

Consider that the magnetic fluid layer in an initial equilibrium state will be perturbed by the input magnetic field applied at the bottom of the layer, the perturbation part of the surface dynamic governing equations can then be written as [24]:

(11) $$\begin{array}{cc} \nabla^{2}\phi =0, & -d\leq z\leq \zeta \end{array}$$

(12) $$\begin{array}{cc} \nabla^{2}\psi^{\left(i\right)}=0, & i=1,2,3 \end{array}$$

(13) $$-\rho \frac{\partial \phi }{\partial t}+\rho g\zeta +\chi B_{0}\frac{\partial \psi^{\left(2\right)}}{\partial z}-\sigma \left(\frac{\partial^{2}\zeta }{\partial r^{2}}+\frac{1}{r}\frac{\partial \zeta }{\partial r}+\frac{1}{r^{2}}\frac{\partial^{2}\zeta }{\partial \theta^{2}}\right)=0\;\mathrm{at}\;z=\zeta$$

where ϕ and $\psi^{\left(i\right)}$, i=1,2,3 are the perturbation components of the velocity potential and the magnetic potential, respectively. 

Consider the following two boundary conditions:

(14) $$-\frac{\partial \phi }{\partial z}=\frac{\partial \zeta }{\partial t}\;\mathrm{at}\;z=\zeta$$

(15) $$-\frac{\partial \phi }{\partial z}=0\;\mathrm{at}\;z=-d$$

the solution of ϕ then can be solved as:

(16) $$\phi \left(r,\theta ,z,t\right)=-\frac{1}{\lambda }\frac{\cosh\left[\lambda \left(z+d\right)\right]}{\sinh\left(\lambda d\right)}\frac{d\tilde{\zeta }}{dt}R\left(t\right)\Theta \left(\theta \right)$$

where λ is the separation constant, and Θ(θ) and R(r) satisfy the following ordinary differential equations:

(17) $$\frac{d^{2}\Theta }{d\theta^{2}}+m^{2}\Theta =0$$

(18) $$\left(\frac{d^{2}R}{dr^{2}}+\frac{1}{r}\frac{dR}{dr}\right)+\left(\lambda^{2}-\frac{m^{2}}{r^{2}}\right)R=0$$

where λ is yet another separation constant.

Based on the magnetic field boundary conditions between the three different materials and considering the magnetic potential sources of input magnetic coils as follows:

(19) $$\underset{z\to \infty }{\lim}\psi^{\left(1\right)}<\infty$$

(20) $$\hat{n}\times \left(H^{\left(2\right)}-H^{\left(1\right)}\right)=0\;\mathrm{at}\;z=\zeta$$

(21) $$\hat{n}\cdot \left(B^{\left(2\right)}-B^{\left(1\right)}\right)=0\;\mathrm{at}\;z=\zeta$$

(22) $$\hat{z}\times \left(H^{\left(3\right)}-H^{\left(2\right)}\right)=0\;\mathrm{at}\;z=-d$$

(23) $$\hat{z}\cdot \left(B^{\left(3\right)}-B^{\left(2\right)}\right)=0\;\mathrm{at}\;z=-d$$

(24) $$\psi^{\left(3\right)}\left(r,\theta ,z,t\right)=\sum_{j=1}^{J}\psi_{j}\left(t\right)\frac{1}{r}\delta \left(r-r_{j}\right)\delta \left(\theta -\theta_{j}\right)\;\mathrm{at}\;z=-h$$

The $\psi^{\left(i\right)}$, i=1,2,3, in Equation (12) can be further solved as:

(25) $$\psi^{\left(1\right)}\left(r,\theta ,z,t\right)=-A\left(t\right)\frac{\mu }{\mu_{0}}e^{-\lambda z}R\left(r\right)\Theta \left(\theta \right)$$

(26) $$\psi^{\left(2\right)}\left(r,\theta ,z,t\right)=-\left(A\left(t\right)X\left(\lambda z\right)+\frac{\chi }{\mu_{0}}B_{0}\tilde{\zeta }\left(t\right)\cosh\left(\lambda z\right)\right)R\left(r\right)\Theta \left(\theta \right)$$

(27) $$\psi^{\left(3\right)}\left(r,\theta ,z,t\right)=\left(A\left(t\right)Y\left(\lambda z\right)-Z\left(\lambda z\right)B_{0}\tilde{\zeta }\left(t\right)\right)R\left(r\right)\Theta \left(\theta \right)$$

where A(t) is the integration constant, and

(28) $$X\left(\lambda z\right)=\frac{\mu }{\mu_{0}}\cosh\left(\lambda z\right)-\sinh\left(\lambda z\right)$$

(29) $$Y\left(\lambda z\right)=-\left(\frac{\mu }{\mu_{0}}\frac{\beta }{\alpha }+\frac{\chi }{\alpha }\right)\cosh\left(\lambda z\right)+\left(\frac{\mu }{\mu_{0}}\left(\frac{\alpha }{\beta }-\frac{\chi }{\alpha }\right)-\frac{\chi^{2}}{\alpha \beta }\right)\sinh\left(\lambda z\right)$$

(30) $$Z\left(\lambda z\right)=\left(\beta \cosh\left(\lambda z\right)+\chi \sinh\left(\lambda z\right)\right)\frac{\chi }{\mu }\frac{1}{\alpha }$$

(31) α=tanh(λd)−coth(λd)

(32) $$\beta =\frac{\mu }{\mu_{0}}\tanh\left(\lambda d\right)-\coth\left(\lambda d\right)$$

(33) $$\Theta \left(\theta \right)=\{\begin{array}{cc} \sin m\theta , & m=1,2,3,\ldots \\ \cos m\theta , & m=0,1,2,\ldots \end{array}$$

(34) $$R\left(r\right)=CJ_{m}\left(\lambda r\right)$$

C is the constant of integration and $J_{m}\left(\cdot \right)$ is the Bessel function of the first kind.

Considering that the miniature coils are located far from the walls of the fluid container, we have $J_{m}\left(\lambda R\right)=0$, at r=R, providing the eigenvalue $\lambda_{mn}$ for each mode as $\lambda_{mn}=\varepsilon_{mn}/R$. Combing Equations (33) and (34), the mode shapes of $H_{mnc}=J_{m}\left(\lambda_{mn}r\right)\cos\left(m\theta \right)$ and $H_{mns}=J_{m}\left(\lambda_{mn}r\right)\sin\left(m\theta \right)$ are obtained.

With the derived ϕ, ψ, and Equation (13), the following surface dynamic equation with respect to the mode shape $H_{mnc}$ is obtained as:

(35) $$\frac{d^{2}{\tilde{\zeta }}_{mnc}\left(t\right)}{dt^{2}}+\omega_{d_{mn}}\frac{d{\tilde{\zeta }}_{mnc}\left(t\right)}{dt}+\omega_{mn}^{2}{\tilde{\zeta }}_{mnc}\left(t\right)=f_{mnc}\left(t\right)$$

where

(36) $$\omega_{mn}^{2}=g\tanh\left(\lambda_{mn}d\right)\lambda_{mn}+\frac{\sigma }{\rho }\tanh\left(\lambda_{mn}d\right)\lambda_{mn}^{3}+\frac{\chi }{\rho }B_{0}^{2}\tanh\left(\lambda_{mn}d\right)\lambda_{mn}^{2}\frac{Z\left(-\lambda_{mn}h\right)}{Y\left(-\lambda_{mn}h\right)}$$

(37) $$\omega_{d_{mn}}=4\frac{\eta }{\rho }\lambda_{mn}^{2}$$

(38) $$f_{mnc}\left(t\right)=F_{mn}\sum_{j=1}^{J}\psi_{j}\left(t\right)H_{mnc}^{j}$$

(39) $$F_{mn}=-\frac{\chi }{p}B_{0}\frac{\tanh\left(\lambda_{mn}d\right)}{Y\left(-\lambda_{mn}h\right)}\lambda_{mn}^{2}\frac{k}{\pi R^{2}{\left[J_{m+1}\left(\varepsilon_{mn}\right)\right]}^{2}}$$

(40) $$H_{mnc}^{j}=J_{m}\left(\lambda_{mn}r_{j}\right)\cos\left(m\theta_{j}\right)$$

(41) $$\begin{array}{ccc} m=0,1,2,\ldots , & n=1,2,3,\ldots , & j=1,2,3,\ldots ,J \end{array}$$

Similarly, the surface dynamic equation with respect to the mode shape $H_{mns}$ is also obtained as:

(42) $$\frac{d^{2}{\tilde{\zeta }}_{mns}\left(t\right)}{dt^{2}}+\omega_{d_{mn}}\frac{d{\tilde{\zeta }}_{mns}\left(t\right)}{dt}+\omega_{mn}^{2}{\tilde{\zeta }}_{mns}\left(t\right)=f_{mns}\left(t\right)$$

where

(43) $$f_{mns}\left(t\right)=F_{mn}\sum_{j=1}^{J}\psi_{j}\left(t\right)H_{mns}^{j}$$

(44) $$H_{mns}^{j}=J_{m}\left(\lambda_{mn}r_{j}\right)\sin\left(m\theta_{j}\right)$$

Based on the ${\tilde{\zeta }}_{mnc}\left(t\right)$, $H_{mnc}$ and ${\tilde{\zeta }}_{mns}\left(t\right)$, $H_{mns}$, the total surface displacement at any desired location $\left(r_{k},\theta_{k}\right)$ is then given as:

(45) $$\zeta \left(r_{k},\theta_{k},t\right)=\sum_{m=0}^{\infty }\sum_{n=1}^{\infty }{\tilde{\zeta }}_{mnc}\left(t\right)H_{mnc}\left(r_{k},\theta_{k}\right)+\sum_{m=1}^{\infty }\sum_{n=1}^{\infty }{\tilde{\zeta }}_{mns}\left(t\right)H_{mns}\left(r_{k},\theta_{k}\right)$$

Based on Equations (35), (42) and (45), the state-space model of the mirror can be further written as:

(46) $$\begin{array}{l} \dot{x}=Ax+B^{\prime }u^{\prime }\\ y=Cx \end{array}$$

where $x={\left[{\tilde{\zeta }}_{01}{\dot{\tilde{\zeta }}}_{01}\cdots {\tilde{\zeta }}_{0N}{\dot{\tilde{\zeta }}}_{0N}{\tilde{\zeta }}_{11c}{\dot{\tilde{\zeta }}}_{11c}{\tilde{\zeta }}_{11s}{\dot{\tilde{\zeta }}}_{11s}\cdots {\tilde{\zeta }}_{MNc}{\dot{\tilde{\zeta }}}_{MNc}{\tilde{\zeta }}_{MNs}{\dot{\tilde{\zeta }}}_{MNs}\right]}_{\left(1\times 2N(2M+1)\right)}^{T}$ is the vector of the generalized displacements and the corresponding velocities, $u^{\prime }={\left[\psi_{1}\psi_{2}\cdots \psi_{J}\right]}_{\left(1\times J\right)}^{T}$ is the vector of input magnetic potentials, and $y={\left[\zeta_{1}\zeta_{2}\zeta_{3}\cdots \zeta_{K}\right]}_{\left(1\times K\right)}^{T}$ is the vector of wavefront produced by the deformable mirror at K sampling points. A, $B^{\prime }$ and C are the corresponding system matrices. If ϑ is the slope of the current-potential relationship, then $u^{\prime }=\vartheta U$ $\left(B=\vartheta B^{\prime }\right)$, and a discrete-time equivalent representation of model is given by

(47) $$\begin{array}{l} x\left(k+1\right)=A_{d}x\left(k\right)+B_{d}U\\ y\left(k\right)=C_{d}x\left(k\right) \end{array}$$

where x(k) is the vector of state variables, U is the vector of control currents, y(k) is the vector of the mirror surface deflections, $A_{d}{=e}^{TA}$ is the system matrices, $B_{d}=\int_{0}^{T}e^{\tau A}d\tau B$ is the input matrices, $C_{d}=C$ is the output matrices and T is the sampling period.

The direct current (DC) gain of the system relating the steady-state response to the input current can be obtained as

(48) $$G=C_{d}{\left(I-A_{d}\right)}^{-1}B_{d}$$

The DC gain of the system can also be represented as the following influence matrix w.r.t each actuator:

(49) $$G={\left[\begin{array}{cccc} G_{1}\left(P_{1}\right) & G_{2}\left(P_{1}\right) & \cdots & G_{J}\left(P_{1}\right)\\ G_{1}\left(P_{2}\right) & G_{2}\left(P_{2}\right) & \cdots & G_{J}\left(P_{2}\right)\\ \vdots & \vdots & \ddots & \vdots \\ G_{1}\left(P_{K}\right) & G_{2}\left(P_{K}\right) & \cdots & G_{J}\left(P_{K}\right) \end{array}\right]}_{K\times J}$$

where $P_{k}$ represents the $k^{\mathrm{th}}$ sampling point (k=1,2,…,K).

## 3. The Model-Based Control Algorithm

### 3.1. Relationship Between the Second Moment of the Aberration Gradients and the Far-Field Intensity Distribution

The centroid of the far-field intensity distribution in geometric optics is related to the aberration of the input wavefront, which can be described as follows [21]:

(50) $$SM\approx c_{0}\left(1-MDS\right)$$

where $c_{0}$ is a tunable parameter. On the left-hand side of Equation (50), SM represents the second moment of the aberration gradients that is defined as

(51) $$SM=\frac{\iint_{s}\left\{{\left[\frac{\partial }{\partial x}W\left(x,y\right)\right]}^{2}+{\left[\frac{\partial }{\partial y}W\left(x,y\right)\right]}^{2}\right\}dxdy}{s}$$

where W(x,y) stands for the aberration, s is the area of incident light pupil plane whose coordinate is indicated as (x,y). At the right-hand side of Equation (50), MDS is the signal of optics intensity that can be defined as follows:

(52) $$MDS=\frac{\int_{x^{\prime }}\int_{y^{\prime }}I\left(x^{\prime },y^{\prime }\right)\left[1-\frac{{r^{\prime }}^{2}}{{R^{\prime }}^{2}}\right]dx^{\prime }dy^{\prime }}{\int_{x^{\prime }}\int_{y^{\prime }}I\left(x^{\prime },y^{\prime }\right)dx^{\prime }dy^{\prime }}$$

where $I(x^{\prime },y^{\prime })$ is the far-filed intensity at $\left(x^{\prime },y^{\prime }\right)$, $r^{\prime }=\sqrt{{x^{\prime }}^{2}+{y^{\prime }}^{2}}$ is a suitable chosen CCD radius, and $R^{\prime }$ is weighted by the system’s diffraction limitation. According to the relationship introduced by Equation (50), we will build the general model-based method for the WFSless AO system.

In order to find out the control input for the magnetic fluid deformable mirror, Z orthogonal modes are taken as the predetermined bias functions and are added by the MFDM sequentially with coefficient δ to the wavefront aberration that needs be corrected. Then the far-field optical intensity information is recorded and the $MDS_{i}$ (i=1,…,Z) are calculated according to Equation (52). Zernike parameters V can be estimated by

(53) $$V\approx \frac{S^{-1}\left(c_{0}*M-\delta^{2}*S_{z}\right)}{2*\delta }$$

where

(54) $$M=-\left(\begin{array}{c} MDS_{1}-MDS_{0}\\ MDS_{2}-MDS_{0}\\ \cdots \\ MDS_{Z}-MDS_{0} \end{array}\right)$$

and $MDS_{0}$ is the corresponding MDS of the wavefront aberration to be corrected. The matrix S is the second moment of the wavefront gradients, which can be calculated by the bias function. The vector $S_{z}$ is the diagonal vector of matrix S. $W\left(x,y\right)=\sum_{i=1}^{Z}v_{i}F_{i}\left(x,y\right)$ represents the aberration of the wavefront that can be expressed by a series of Z orthonormal modes $F_{i}\left(x,y\right)$. Then S can be calculated according to the following equation:

(55) $$S\left(i,q\right)=\frac{\iint_{s}\left\{\left[\frac{\partial }{\partial x}F_{i}\left(x,y\right)\frac{\partial }{\partial x}F_{q}\left(x,y\right)\right]+\left[\frac{\partial }{\partial y}F_{i}\left(x,y\right)\frac{\partial }{\partial y}F_{q}\left(x,y\right)\right]\right\}dxdy}{s}$$

### 3.2. Control Algorithm

According to the relationship between the MDS and the SM of the aberration gradient, the model-based WFSless control algorithm for the MFDM is developed in the following sections. 

#### 3.2.1. Preprocessing

**Figure 2:**
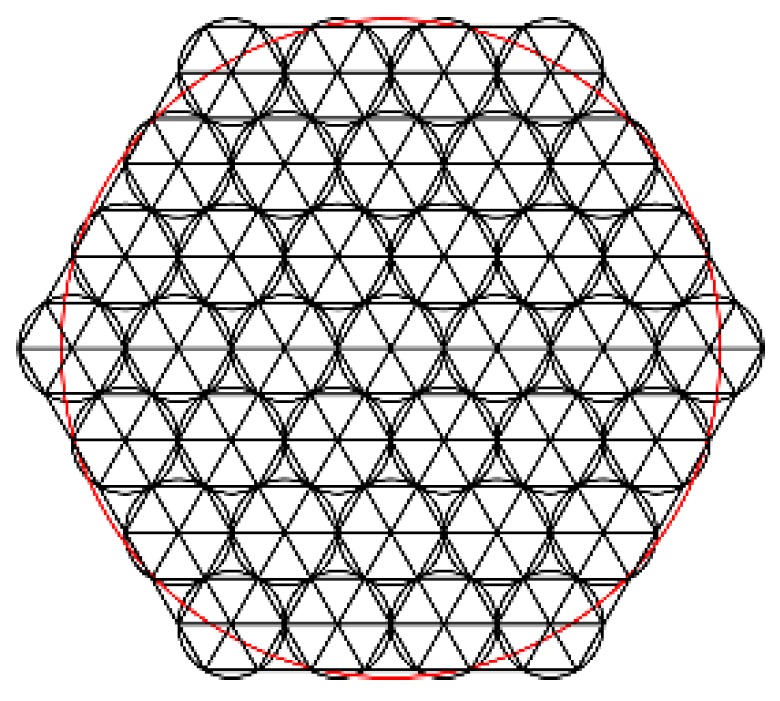
The arrangement of electromagnetic actuators of magnetic fluid deformable mirror (MFDM).

Figure 2 shows the arrangement of electromagnetic actuators of MFDM. The sampling points are the center of each triangle that makes up the pupil.

With respect to K sampling points at the mirror surface, the wavefront aberrations at each sampling point can be written as

(56) $$\begin{array}{cc} W\left(P_{k}\right)=\sum_{i=1}^{Z}v_{i}F_{i}\left(P_{k}\right), & k=1,2,\ldots ,K \end{array}$$

Define the Zernike function matrix as:

(57) $$F={\left[\begin{array}{cccc} F_{1}\left(P_{1}\right) & F_{2}\left(P_{1}\right) & \cdots & F_{Z}\left(P_{1}\right)\\ F_{1}\left(P_{2}\right) & F_{2}\left(P_{2}\right) & \cdots & F_{Z}\left(P_{2}\right)\\ \vdots & \vdots & \ddots & \vdots \\ F_{1}\left(P_{K}\right) & F_{2}\left(P_{K}\right) & \cdots & F_{Z}\left(P_{K}\right) \end{array}\right]}_{K\times Z}$$

Using MFDM to fit the wavefront aberration of Equation (56) described by Zernike polynomials, we can get:

(58) $$\sum_{i=1}^{Z}v_{i}F_{i}\left(P_{k}\right)=\sum_{j=1}^{J}u_{j}G_{j}\left(P_{k}\right)+E$$

where $u_{j}$ is the voltage of $j^{\mathrm{th}}$ actuators, and E is the wavefront residual error. Multiply both sides of Equation (58) by the influence matrix $G^{T}$, and then calculate the average integral on s, which can be written as:

(59) $$C_{zv}V=C_{v}U+\varepsilon$$

(60) $$C_{zv}=\frac{\frac{s}{K}\times G^{T}F}{s}=\frac{G^{T}F}{K}$$

(61) $$C_{v}=\frac{\frac{s}{K}\times G^{T}G}{s}=\frac{G^{T}G}{K}$$

where ε is the vector of the residual error, $\frac{s}{K}$ is the sampling area of each triangle, $C_{zv}$ is the matrix of the relationship between the influence functions and the Zernike polynomials, $C_{v}$ is the coupling matrix between the influence functions that is symmetric reversible. Then, the optimal least-squares solution of Equation (59) is formulated as:

(62) $$U^{*}=C_{v}{}^{-1}C_{zv}V$$

#### 3.2.2. Iterative algorithm

A closed-loop algorithm based on the developed model is used to control the deformation of MFDM in the WFSless AO system. The algorithm is described as follows:

(1) Gather the corresponding far-field intensity with the wavefront W(x,y) from CCD, then calculate $MDS_{0}$ by Equation (52).
(2) For each step i=1,⋯,Z, denote $V_{i}={\left[\begin{array}{ccccc} 0 & \cdots & \delta & \cdots & 0 \end{array}\right]}^{T}$, where only the $i^{\mathrm{th}}$ element of the row is δ and the others are zero. Bring the coefficient $V_{i}$ into the Equation (62) to obtain the optimal voltage for each Zernike mode and apply the voltage vector to the actuators of the MFDM. The wavefront shape introduced by the MFDM is then superimposed to the wavefront W(x,y). Detect the far-field optical intensity of the modified wavefront aberration from CCD and then calculate $MDS_{i}$ according to Equation (52).
(3) Repeating (2), obtain $MDS_{1},MDS_{2},\cdots ,MDS_{Z}$ for each Zernike mode, respectively.
(4) Compute M according to Equation (54).
(5) Obtain the corresponding Zernike parameters V of the wavefront aberration based on Equation (53). Plug the control parameters V into Equation (62) to obtain the voltage vector U applied to the actuators of MFDM.
(6) Regarding the residual wavefront aberration, repeat the iterative step (1)–(5) until the algorithm satisfies the termination conditions, such as certain number of iterations.

Compared with other WFSless control methods, the above model-based WFSless closed-loop control algorithm needs fewer steps and has better convergence performance.

## 4. Experimental Verification

### 4.1. Experiment Setup

**Figure 3:**
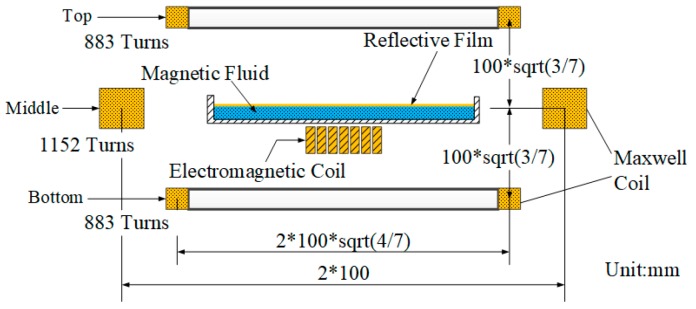
The schematic diagram of the MFDM.

**Figure 4:**
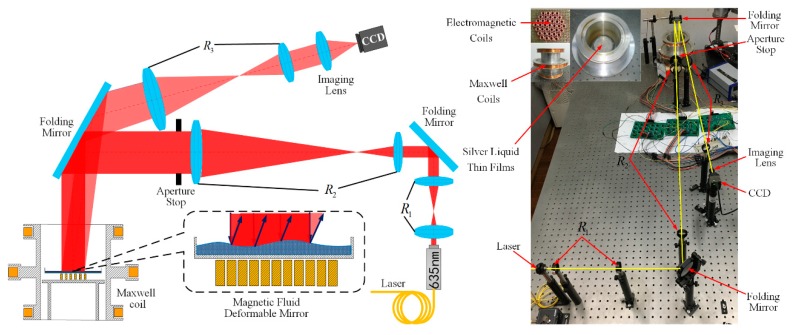
The schematic and actual layout of the experimental setup of wavefront sensorless (WFSless) adaptive optics (AO) system.

The properties of the magnetic fluid used in this paper are given in Table 1. In order to verify the performance of the correction ability of the MFDM, Figure 3 shows the schematic diagram of the designed MFDM that consists of the magnetic fluid in container, a thin film of a reflective material coated on the free surface of the magnetic fluid, miniature electromagnetic coils placed underneath the fluid layer, and a Maxwell coil. Figure 4 illustrates the schematic and actual layout of the experimental setup of the WFSless AO system. Components of the setup have been labeled along with the path of the laser beam. The 635 nm laser beam is expanded through the first and second optic relays R1, R2 and an optic aperture until it is deflected down to the horizontal MFDM by the folding mirror. The reflected beam will reflect directly back onto the folding mirror and the third optic relay R3 which minifies the diameter of the laser beam. The laser beam is focused on the CCD camera by the imaging lens. The CCD camera (DCU223C, Thorlabs) is used to image the geometric profile and measure the intensity profile of the beam.

### 4.2. Experimental Results

To evaluate the correction capabilities of the MFDM with the proposed model-based WFSless control algorithm in the AO system, $MDS_{0}$ and Strehl ratio (SR) are used to measure the correction capability of the AO system, SR is defined as

(63) $$SR=\frac{P\left[I\left(x^{\prime },y^{\prime }\right)\right]}{P\left[I_{0}\left(x^{\prime },y^{\prime }\right)\right]}$$

where P[⋅] is an operation, which calculates the peak intensity. $I_{0}\left(x^{\prime },y^{\prime }\right)$ is the intensity distribution without aberration. $MDS_{0}$ based on Equation (52) is defined as the value of MDS before correction.

**Figure 5:**
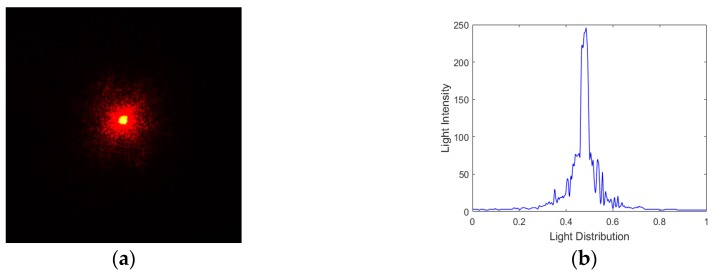
(**a**) The focal spot image captured by Charge-coupled Device (CCD) without aberration disturbance; (**b**) light intensity distribution

Figure 5a shows the desired focal spot of the laser beam captured by a CCD camera without the aberration disturbance in the optical path, and the light intensity distribution is shown in Figure 5b. Firstly, the inverse matrix $S^{-1}$, vector $S_{z}$ (diagonal vector of matrix S), matrix $C_{zv}$ and matrix $C_{v}$ are calculated once and for all. The approximate linear relation constant is $c_{0}=0.023$ and the parameter δ is set to 0.16.

**Figure 6:**
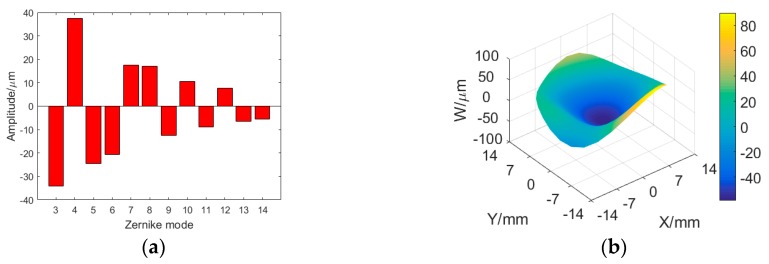
Target aberration. (**a**) Zernike mode numbers of the target aberration; (**b**) the aberration wavefront.

**Figure 7:**
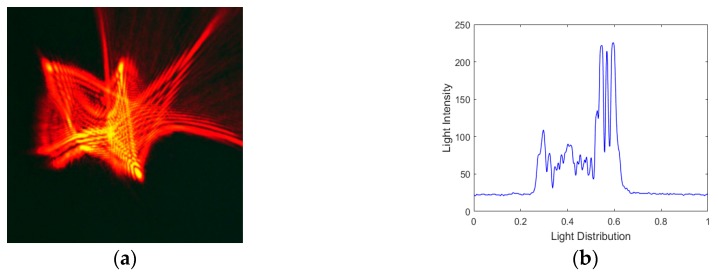
(**a**) The image captured by CCD with random aberration generated by the MFDM; (**b**) light intensity distribution.

Secondly, an unknown random aberration is generated by applying a vector of 37 random control signals to the actuators of the magnetic fluid deformable mirror, the corresponding Zernike mode numbers and the resulting wavefront are shown in Figure 6. The laser beam is incident on the MFDM with this aberration, and the optical intensity profile distribution after reflection has been shown in Figure 7. We can see the laser beam become disorderly.

**Figure 8:**
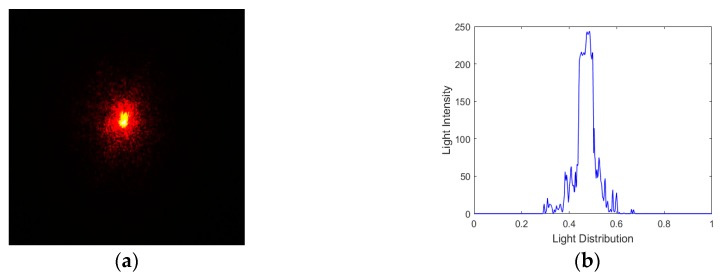
(**a**) The image captured by CCD after correction; (**b**) light intensity distribution.

**Figure 9:**
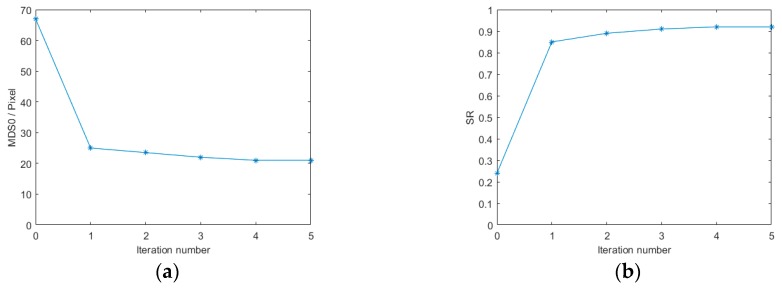
The trend of the performance metric (**a**) $MDS_{0}$ curve; (**b**) Sterhl ratio (SR) curve.

Finally, 12 (3–14) modes with the parameter δ were taken sequentially to be the biases of the model-based control method and added by the MFDM to the input wavefront. According to Equations (53) and (62), the closed-loop control algorithm computes the ideal control signal vector needed for correction of the random aberration and then applies to the MFDM to eliminate the large unknown aberration. After the first iteration, the $MDS_{0}$ drops from 67.34 to 24.79 and SR rises from 0.24 to 0.87. In order to further verify the performance of the WFSless control algorithm, several iterations have been implemented. The final result after five corrections captured by the CCD has been shown in Figure 8, where the divergent spot has been concentrated to the focal spot. Meanwhile, Figure 9 shows the trend of the performance metric by the control algorithm after the five iterations, where the final $MDS_{0}$ is equal to 21.37 and the final SR is 0.92. It can be seen that the curves of different metrics have converged after five iterations and the one iteration can almost make the laser beam converge in the AO system with MFDM. Thus, the unknown aberration has been effectively corrected by the MFDM with the proposed WFSless control algorithm in the AO system. 

## 5. Conclusions

This paper presented a model-based WFSless control method for the magnetic fluid deformable mirror to compensate for the unknown large aberrations in the AO system. According to the established surface dynamics model of the MFDM, the model-based control approach is developed based on a mapping relationship between the second moments of the wavefront gradients and the far-field intensity distribution by taking Zernike polynomials as the predetermined bias functions. The unknown aberrations can be corrected without the wavefront measurement in the closed-loop AO control system. A WFSless AO experiment platform with a prototype MFDM is also setup for the experimental evaluation. Experiment results indicate that the MFDM can be effectively controlled by the model-based WFSless control approach to produce the desired surface deformation for the unknown aberration elimination in the imaging system.

## Figures and Tables

**Table 1:** Parameters of the magnetic fluid.

Magnetic Fluid	Parameters
Saturation magnetization	22 mT
Relative permeability	2.89
Density	1190 kg/m^3^
Viscosity	3 cP
Thickness	1 mm
